# COVCOG 1: Factors Predicting Physical, Neurological and Cognitive Symptoms in Long COVID in a Community Sample. A First Publication From the COVID and Cognition Study

**DOI:** 10.3389/fnagi.2022.804922

**Published:** 2022-03-17

**Authors:** Panyuan Guo, Alvaro Benito Ballesteros, Sabine P. Yeung, Ruby Liu, Arka Saha, Lyn Curtis, Muzaffer Kaser, Mark P. Haggard, Lucy G. Cheke

**Affiliations:** ^1^Department of Psychology, University of Cambridge, Cambridge, United Kingdom; ^2^School of Psychology, College of Life and Environmental Sciences, University of Exeter, Exeter, United Kingdom; ^3^Department of Psychiatry, University of Cambridge, Cambridge, United Kingdom; ^4^Cambridgeshire and Peterborough NHS Foundation Trust, Cambridge, United Kingdom

**Keywords:** Long COVID, cognition, neurological, memory, executive functions, language, COVID-19, symptoms

## Abstract

Since its first emergence in December 2019, coronavirus disease 2019 (COVID-19), caused by severe acute respiratory syndrome coronavirus 2 (SARS-CoV-2), has evolved into a global pandemic. Whilst often considered a respiratory disease, a large proportion of COVID-19 patients report neurological symptoms, and there is accumulating evidence for neural damage in some individuals, with recent studies suggesting loss of gray matter in multiple regions, particularly in the left hemisphere. There are a number of mechanisms by which COVID-19 infection may lead to neurological symptoms and structural and functional changes in the brain, and it is reasonable to expect that many of these may translate into cognitive problems. Indeed, cognitive problems are one of the most commonly reported symptoms in those experiencing “Long COVID”—the chronic illness following COVID-19 infection that affects between 10 and 25% of patients. The COVID and Cognition Study is a part cross-sectional, part longitudinal, study documenting and aiming to understand the cognitive problems in Long COVID. In this first paper from the study, we document the characteristics of our sample of 181 individuals who had experienced COVID-19 infection, and 185 who had not. We explore which factors may be predictive of ongoing symptoms and their severity, as well as conducting an in-depth analysis of symptom profiles. Finally, we explore which factors predict the presence and severity of cognitive symptoms, both throughout the ongoing illness and at the time of testing. The main finding from this first analysis is that that severity of initial illness is a significant predictor of the presence and severity of ongoing symptoms, and that some symptoms during the initial illness—particularly limb weakness—may be more common in those that have more severe ongoing symptoms. Symptom profiles can be well described in terms of 5 or 6 factors, reflecting the variety of this highly heterogenous condition experienced by the individual. Specifically, we found that neurological/psychiatric and fatigue/mixed symptoms during the initial illness, and that neurological, gastrointestinal, and cardiopulmonary/fatigue symptoms during the ongoing illness, predicted experience of cognitive symptoms.

## Introduction

Manifestations of coronavirus 2 (SARS-CoV-2) infection vary in severity ranging from asymptomatic to fatal. In the acute stage, symptomatic patients—at least in the early variants—typically experience respiratory difficulties that can result in hospitalization and require assisted ventilation (Baj et al., 2020; Heneka et al., 2020; Jain, 2020). While COVID-19 is primarily associated with respiratory and pulmonary challenge, 35% of patients report neurological symptoms including headache and dizziness (e.g., Mao et al., 2020). In severe illness, neurological symptoms can be seen in 50–85% of patients (e.g., Pryce-Roberts et al., 2020; Romero-Sánchez et al., 2020). Indeed, alteration in taste or smell (anosmia/dysgeusia) is reported in over 80% of cases (e.g., Lechien et al., 2020), is often the first clinical symptom (Mao et al., 2020; Romero-Sánchez et al., 2020) and regularly persists beyond resolution of respiratory illness (Lechien et al., 2020).

Accumulating evidence suggests that many COVID-19 patients experiencing severe illness show evidence of neural damage (Helms et al., 2020; Kandemirli et al., 2020) and unusual neural activity (Galanopoulou et al., 2020). There are a number of postulated mechanisms linking COVID-19 infection with neurological problems (Bougakov et al., 2021). For example, based on the behavior of previous SARS viruses, SARS-CoV-2 may attack the brain directly perhaps via the olfactory nerve (Lechien et al., 2020; Politi et al., 2020) causing encephalitis. Severe hypoxia from respiratory failure or distress can also induce hypoxic/anoxic-related encephalopathy (Guo et al., 2020). There is considerable evidence that COVID-19 is associated with abnormal blood coagulation, which can increase risk of acute ischemic and hemorrhagic cerebrovascular events (CVAs) (Beyrouti et al., 2020; Li et al., 2020; Wang et al., 2020; Kubánková et al., 2021) leading to more lasting brain lesions. Indeed, ischemic or hemorrhagic lesions have been found in COVID-19 patients in multiple studies (Le Guennec et al., 2020; Matschke et al., 2020; Moriguchi et al., 2020; Poyiadji et al., 2020). A recent study using the United Kingdom Biobank cohort comparing structural and functional brain scans before and after infection with COVID-19 identified significant loss of gray matter in the parahippocampal gyrus, lateral orbitofrontal cortex and insula, notably concentrated in the left hemisphere in patients relative to controls (Douaud et al., 2021).

A key candidate mechanism is dysfunctional or excessive immune response to infection. For example, excessive cytokine release (“cytokine storm”) and immune-mediated peripheral neuropathy (e.g., Guillain-Barre syndrome) are both linked with neurological and sensory-motor issues (Alberti et al., 2020; Das et al., 2020; Poyiadji et al., 2020; Whittaker et al., 2020; Zhao et al., 2020). In addition to acute effects, chronic inflammation has also been associated with neural and cognitive dysfunction, particularly in the hippocampus—a key area responsible for memory (Ekdahl et al., 2003; Monje et al., 2003; Jakubs et al., 2008; Belarbi et al., 2012). Considerable rodent evidence links inflammatory cytokines with cognitive impairments (e.g., IL-1β: Thirumangalakudi et al., 2008; Beilharz et al., 2014, 2018; Che et al., 2018; Mirzaei et al., 2018; TNF-α: Thirumangalakudi et al., 2008; Beilharz et al., 2014; Almeida-Suhett et al., 2017). These findings are broadly reflected in human studies, wherein circulating cytokines have been associated with reduced episodic memory (e.g., Kheirouri and Alizadeh, 2019) and chronic neuroinflammation has been heavily implicated in the pathophysiology of neurodegenerative diseases (McGeer and McGeer, 2010; Zotova et al., 2010; Chen et al., 2016; Bossù et al., 2020). Given the volume of reports of excessive immune response to COVID-19 infection (Mehta et al., 2020; Tay et al., 2020), and evidence for neuroinflammation from postmortem reports (Matschke et al., 2020) research into cognitive sequalae is highly implicated.

Given the evidence for widespread neural symptoms and demonstrable neural damage, it could be expected that COVID-19 infection would be associated with cognitive deficits. Indeed, there is some early evidence linking neural changes following COVID-19 and cognitive deficits. Hosp et al. (2021) found that evidence of frontoparietal hypometabolism in older patients presenting with post-COVID-19 neurological symptoms via positron emission tomography (PET) was associated with lower neuropsychological scores, particularly in tests of verbal memory and executive functions.

Many forms of neuropathology would be unlikely to be present uniquely as cognitive deficits, but would be associated with a range of related symptoms. Some of these symptoms may be neurological (e.g., disorientation, headache, numbness) while others may reflect systemic/multisystem involvement (e.g., reflecting the symptom profile of chronic inflammatory or autoimmune diseases). It may therefore be possible to gain information as to the mechanism of neurological involvement via investigation of symptomatology. If it is possible to identify groups of symptoms (such as neurological, respiratory, systemic) during either the acute or post-acute phase of illness that predict cognitive problems, this may aid in the identification of patients that are at risk of developing cognitive deficits. In a highly heterogenous condition, in which up to 200 symptoms have been suggested (Davis et al., 2021), reduction of dimensionality is essential to allow meaningful associations to be drawn between experienced symptoms and relevant outcomes.

The United Kingdom Office for National Statistics [ONS] (2021) has estimated that around 21% of those experiencing COVID-19 infection still have symptoms at 5 weeks, and that 10% still have these symptoms at 12 weeks from onset. These figures may not tell the full story, being based on a list of 12 physical symptoms which does not include neurological or cognitive manifestations (e.g., Alwan and Johnson, 2021; Ziauddeen et al., 2021). Other calculations suggest that around 1 in 3 non-hospitalized COVID-19 patients have physical or neurological symptoms after 2–6 weeks from disease onset (Sudre et al., 2020; Tenforde et al., 2020; Nehme et al., 2021) and that 11–24% still have persisting physical, neurological or cognitive symptoms 3 months after disease onset (Cirulli et al., 2020; Ding et al., 2020). A community-based study reported that around 38% symptomatic people experienced at least one physical or neurological symptom lasting 12 weeks or more from onset and around 15% experienced three or more of these symptoms (Whitaker et al., 2021). Ongoing symptoms seem to occur regardless of the severity of the initial infection, with even asymptomatic patients sometimes going on to develop secondary illness (FAIR Health, 2021; Nehme et al., 2021), however, initial severity may impact severity of ongoing issues (e.g., Whitaker et al., 2021).

The National Institute for Health and Care Excellence (NICE) guidelines describe “post-COVID-19 syndrome” as “*Signs or symptoms that develop during or after infection consistent with COVID-19, continue for more than 12 weeks and are not explained by an alternative diagnosis”* (National Institute for Health and Care Excellence [NICE], 2020). One difficulty with this definition is that the “signs or symptoms” that qualify for the diagnosis are not specified (e.g., Alwan and Johnson, 2021; Ziauddeen et al., 2021) thus many patients could go uncounted and unrecognized clinically, or conversely over-liberal inclusion may lead to overcounting. The patient-created term “Long COVID” has increasingly been used as an umbrella term to describe the highly heterogenous condition experienced by many people following COVID-19 infection (Callard and Perego, 2021).

Emerging evidence suggests that Long COVID is a debilitating multisystem illness that affects multiple organ systems and there have been some attempts to characterize “phenotypes.” An online survey involved in 2,550 non-hospitalized participants detected two clusters within both initial and ongoing symptoms. Initial symptoms showed a majority cluster with cardiopulmonary symptoms predominant, and a minority cluster with multisystem symptoms that did not align specifically with any one organ system. Similarly, ongoing symptoms were clustered into a majority cluster with cardiopulmonary, cognitive symptoms and exhaustion, and a minority cluster with multisystem symptoms. Those with more related symptoms in the initial major cluster were more likely to move into ongoing multisystem cluster, and this movement can be predicted by gender and age, with higher risk in women, those younger than 60, and those that took less rest during the initial illness (Ziauddeen et al., 2021).

“Long COVID” research has repeatedly identified cognitive dysfunction as one of the most common persistent symptoms (after fatigue), occurring in around 70% of patients (Cirulli et al., 2020; Bliddal et al., 2021; Davis et al., 2021; Ziauddeen et al., 2021). Indeed, brain fog and difficulty concentrating are more common than cough is at many points in the Long COVID time course (Assaf et al., 2020). Ziauddeen et al. (2021) report nearly 40% of participants endorsing at least one cognitive symptom during the initial 2 weeks of illness, with this persisting in the long term. However around 30% of participants also reported developing cognitive symptoms—particularly brain fog and memory problems—later. Indeed, Davis et al. (2021) demonstrate that brain fog, memory problems and speech and language problems were more commonly reported at week 8 and beyond than they were during initial infection. Furthermore, strenuous cognitive activity was found to be one of the most common triggers leading to relapse/exacerbation of existing symptoms (Davis et al., 2021; Ziauddeen et al., 2021). Crucially, 86% of participants indicated that cognitive dysfunction and/or memory impairment was impacting their ability to work, with nearly 30% reporting being “severely unable to work” and only 27% working as many hours as they had pre-COVID-19 (Davis et al., 2021). These figures suggest that the cognitive sequelae of COVID-19 have the potential for long-term consequences not just for individuals but also—given the prevalence of Long COVID—for the economy and wider society.

Here we report on the first stage of a mixed cross-sectional/longitudinal investigation—The COVID and Cognition Study (COVCOG)—aimed at understanding cognition in post-acute COVID-19. The aims of this current paper are threefold: First, to provide a detailed demographic profile of our sample, comparing those who had experienced COVID-19 infection to those who had not, and those who recovered to those who continued to experience COVID-19 symptoms after acute phase of illness. Second, we aim to contribute to the understanding of phenotypes of Long COVID by using a rigorous factor analytic approach to identify groups of symptoms that tend to co-occur. We investigate symptom profiles both during and following initial infection in those that had experienced COVID-19. This allows investigation of symptoms during initial illness that may be predictive of ongoing symptoms, as well as exploring the nature of those ongoing symptoms themselves. These phenotypes may, through future studies, be directly linked to disease profiles and mechanisms. In an application of this second aim, a third objective is to use the symptom factors extracted (such as those incorporating neurological symptoms) to investigate predictors of self-reported cognitive deficits. Due to the novel character of both the virus and the subsequent ongoing illness at the time of study creation, this study was designed not to test specific hypotheses but to map the terrain, generating hypotheses for future, more targeted investigation.

## Materials and Methods

### Participants

A total of 421 participants aged 18 and over were recruited through word of mouth, student societies and online/social media platforms such as the Facebook *Long COVID Support Group* (over 40K members). Of these, 163 participants were recruited through the *Prolific* recruitment site, targeting participants with demographic profiles otherwise underrepresented in our sample. Specifically, recruitment through *Prolific* was limited to those with low socioeconomic status and levels of education below a bachelor’s degree. As the study was conducted in English, participants were recruited from majority English speaking countries (the United Kingdom, Ireland, United States, Canada, Australia, New Zealand, or South Africa). Informed consent to use of anonymized data was obtained prior to starting.

Data collection for this stage of the study took place between October 2020 and March 2021, and recorded data on infections that occurred between March 2020 and February 2021. As such, all participants with experience of COVID-19 infection were likely to have been infected with either Wild-Type or Alpha-variant SARS-CoV-2, as the later-emerging variants (e.g., Delta, Omicron) were not common in the study countries at that time. Study recruitment started before the roll out of vaccinations, thus we do not have confirmed vaccination status for all participants. Once vaccination became available, the questionnaire was revised to ask about vaccination status. Of the 33 participants who were tested after this point, 11 (2 in the No COVID group, 9 in the COVID group) reported being vaccinated. Among them, 8 had received the first dose and 3 had had two doses. The majority (over 80%) had the vaccine within the last 7 days to last month. All received Pfizer (BNT162b2) except 1 (COVID group) who received AstraZeneca (AZD1222).

### Procedure

The study was reviewed by University of Cambridge Department of Psychology ethics committee (PRE.2020.106, 8/9/2020). The current paper is part of a larger, mixed cross-sectional/longitudinal online study (“COVCOG”) conducted using the online assessment platform Gorilla.^1^ The COVCOG study consists of a baseline assessment of characteristics and cognition in samples of individuals who had or had not experienced COVID-19 infection. Both groups completed questionnaire and a range of cognitive tasks and were then followed up at regular intervals. The results reported here are for the questionnaire section of the baseline session only. The questionnaire covered demographics, previous health and experience of COVID-19.

**FIGURE 1:**
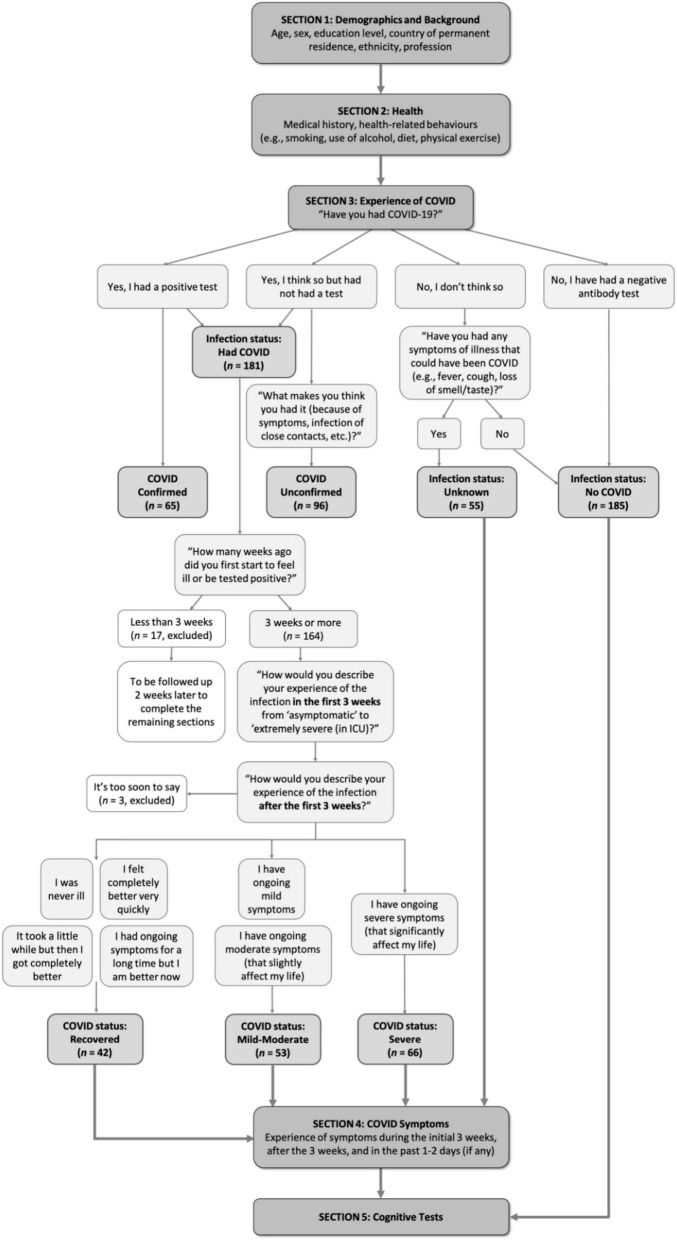
Study procedural flow.

Participants answered questions relating to their age, sex, education level, country of permanent residence, ethnicity, and profession. They were then asked a series of questions relating to their medical history and health-related behaviors. These included self-reporting their height and weight—which were used to calculate body mass index (BMI), and their usual diet intake, use of tobacco and alcohol, and physical activity (before the illness if infected) on a 6-point frequency scale from “Never” to “Several times daily.” Following this, they were asked for details of their experience of COVID-19. Because many of the participants in this study contracted COVID-19 before confirmatory testing of infection state was widely available, both those with (“Confirmed”) and without test confirmation (“Unconfirmed”) were included in the “COVID” group. Those that didn’t think they had had COVID-19 but had experienced an illness that *could* have been COVID-19 were assigned an “Unknown” infection status. Those that confirmed that they had not had COVID-19, nor any illness that might have been COVID-19, were included in the “No COVID” group. The procedure for grouping and progression through the baseline session is detailed in Figure 1.

Participants in the “COVID” group indicated the number of weeks since infection on a drop-down menu. Those that reported being within the first 3 weeks of infection proceeded straight to debriefing and were followed up 2 weeks later, once the initial infection was passed. Apart from this delay, they proceeded with the experiment in the same way as the rest of the COVID group. Participants then answered questions on the severity of the initial illness and whether they were experiencing ongoing symptoms. Finally, participants were asked to give details on a large number of individual symptoms during three time periods: initial illness (first 3 weeks), ongoing illness (“since then,” i.e., the time since initial infection), and currently (past 1–2 days). When reporting on initial symptoms, participants gave an indication of severity on a scale of 1–3 from “Not at all” to “Very severe.” When reporting symptoms over the period “since then” they reported on both severity and regularity of symptoms on a scale of 1–5 from “Not at all” to “Very severe and often.” When reporting on symptoms in the past 1–2 days, they reported the presence or absence of the symptoms dichotomously (i.e., check the box of the symptom if present). These symptom lists were developed based on currently available medical literature reporting symptoms experienced by COVID-19 patients and through consulting medical doctors and COVID-19 patients from the *Long COVID Support Group*. Participants in the “No COVID” Group were not asked their experience of COVID-19.

### Data Processing and Analysis

Analyses were conducted using IBM SPSS Statistics for Windows, Version 23.0. We describe quantitative variables using means and standard deviations, and numbers and percentages for qualitative variables. Sidak’s correction for multiple comparisons was employed. All *p*-values are reported uncorrected, and the Sidak-corrected alpha is quoted where appropriate.

**FIGURE 2:**
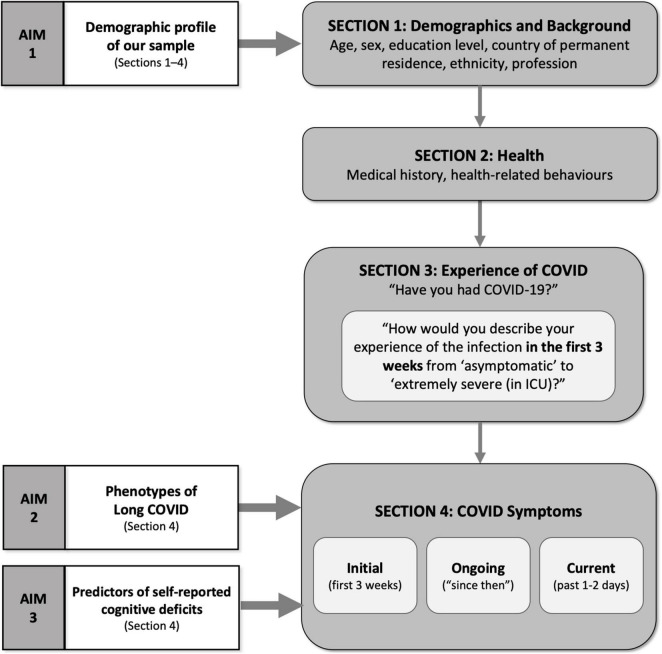
Data analyzed in relation to our study aims.

We investigated differences in the first group of variables: sociodemographic, medical history, and health behaviors, concerning two COVID group classifications. First dividing the sample into two groups (COVID/No COVID), second subdividing the COVID group by symptom longevity and severity (Recovered, Ongoing mild infection, and Ongoing severe infection). Where parametric analysis was not appropriate, we employed the Pearson’s chi-square (χ*^2^*) test for categorical variables and the Mann-Whitney and Kruskal-Wallis test for continuous variables depending on the number of COVID groups. To investigate differences between groups (COVID/No COVID; Recovered/Ongoing mild/Ongoing severe), we employed Mann-Whitney and ANOVA/Kruskal-Wallis. To examine whether these variables and initial symptoms predicted degrees of ongoing illness, we ran independent multinomial logistic regression, using forward stepwise method to identify what items within these variables were significant predictors while controlling for demographics including sex, age, education, and country of residence. Next, to determine suitable groups of symptoms, we employed exploratory principal component analysis (PCA) with varimax rotation. Based on our high number of items (Nunnally, 1978) and the novelty of the subject (Henson and Roberts, 2006), we performed two PCAs, one for the initial symptoms and another one symptoms experienced since the initial phase. We then used the high-loading items on the “since then” symptom factors to calculate profiles for currently experienced symptoms. To explore what symptom factors were associated with infection or ongoing symptoms, we employed various independent multinomial logistic regression with backward elimination of variables *p* > 0.05 to identify the best fitted models. Data analyzed in relation to our study aims are depicted in Figure 2.

## Results

### Sample Characteristics

#### No COVID (*NC*: *n* = 185) vs. COVID (*C*: *n* = 181)

Distributions of demographics including sex, age, education level, country, and ethnicity of the two groups (*NC*/*C*) are shown in Table 1. The majority of participants were from the United Kingdom and were of White (Northern European) ethnicity (over 70% in both groups). Pearson’s chi-square tests showed that the groups did not significantly differ in sex, but differed in age [χ*^2^*(5) = 19.08, *p* = 0.002, *V* = 0.228] and level of education [χ*^2^*(5) = 56.86, *p* < 0.001, *V* = 0.394], with the COVID group tending to fall into the older age ranges and higher education level more than the No COVID group.

**TABLE 1:** Distribution of demographics in No COVID and COVID groups.

	No COVID (*n* = 185)	COVID (*n* = 181)	Chi-square tests
**Sex**			n.s.
Man	63 (34.1%)	48 (26.5%)	
Woman	118 (63.8%)	130 (71.8%)	
Other	4 (2.2%)	3 (1.7%)	
**Age**			χ*^2^*(5) = 19.08, *p* = 0.002, *V* = 0.228
18–20	42 (22.7%)	17 (9.4%)	
21–30	45 (24.3%)	33 (18.2%)	
31–40	37 (20%)	38 (21%)	
41–50	23 (12.4%)	35 (19.3%)	
51–60	25 (13.5%)	39 (21.5%)	
61 or above	13 (7%)	19 (10.5%)	
**Education**			χ*^2^*(5) = 56.86, *p* < 0.001, *V* = 0.394
GCSE or below	20 (10.8%)	14 (7.7%)	
A level	55 (29.7%)	18 (9.9%)	
Attended college without obtaining degree/Technical training/Associate degree	58 (31.4%)	35 (19.3%)	
Bachelor’s degree	21 (11.4%)	55 (30.4%)	
Master’s/Professional degree	17 (9.2%)	49 (27.1%)	
Doctorate degree	14 (7.6%)	10 (5.5%)	
**Country**			n.s.
United Kingdom	137 (74.1%)	130 (71.8%)	
North America	24 (13%)	33 (18.2%)	
Other	24 (13%)	18 (9.9%)	
**Ethnicity**			χ*^2^*(1) = 11.77, *p* = 0.001, *V* = 0.179
Northern European	131 (70.8%)	155 (85.6%)	
Southern European/Latinx	13 (7%)	19 (10.5%)	n.s.
African/Afro-Caribbean	10 (5.5%)	7 (3.9%)	n.s.
Asian	29 (15.6%)	8 (4.5%)	χ*^2^*(1) = 12.76, *p* < 0.001, *V* = 0.187
Other/Prefer not to say	9 (4.8%)	6 (3.4%)	n.s.

#### Employment

Supplementary Table 1 shows the distributions of pre-pandemic profession and employment status. To adjust for multiple comparisons, Sidak corrections were applied and alpha levels were adjusted to 0.003 for profession and 0.007 for employment status. The COVID group had significantly more people working in healthcare [χ*^2^*(1) = 12.77, *p* < 0.001, *V* = 0.187] and engaging in full-time work before the pandemic [χ*^2^*(1) = 21.19, *p* < 0.001, *V* = 0.241]. In contrast, the No COVID group were more likely not to be in paid work [Profession “Not in paid work” χ*^2^*(1) = 27.72, *p* < 0.001, *V* = 0.275; Employment status “Not Working” χ*^2^*(1) = 13.18, *p* < 0.001, *V* = 0.190], and they were more likely to be students [χ*^2^*(1) = 8.91, *p* = 0.003, *V* = 0.156].

#### Health and Medical History

Supplementary Table 2 compares medical history and health behaviors across the COVID and No COVID groups, which may be informative as to vulnerabilities. Sidak correction adjusted the alpha level to 0.003 for medical history and 0.008 for health behaviors. Pearson’s chi-square tests showed that inflammatory or autoimmune diseases [χ*^2^*(1) = 9.81, *p* = 0.002, *V* = 0.164] were found more commonly in the COVID group than the No COVID group. Mann-Whitney *U*-tests showed that the COVID group consumed more fruit and vegetables (*U* = 13,525, *p* = 0.001) and had higher level of physical activity (*U* = 13,752, *p* = 0.002) than the No COVID group, while the No COVID group consumed sugary (*U* = 14168.5, *p* = 0.008) food more than the COVID group. ANOVA showed that the COVID group (*M* = 26.71, *SD* = 7.26) had higher BMI than the No COVID group (*M* = 25.15, *SD* = 5.64), [*F*_(1, 361)_ = 5.24, *p* = 0.023]. However this effect was not significant after controlling for sex, age, education and country [*F*_(1, 357)_ = 1.57, *p* = 0.211].

### Characteristics of Those Experiencing Ongoing Symptoms

To understand the potential association between the progression of COVID-19 and various potential risk factors at baseline, including demographics, medical history and health behaviors, and the severity of initial illness and initial symptoms, we further divided the COVID group into three duration subgroups: (i) those who, at the time of test, had recovered from COVID-19 (“Recovered group,” *R*; *n* = 42), (ii) those who continued to experience mild or moderate ongoing symptoms [“Ongoing (Mild/Moderate) group,” *C* + ; *n* = 53], and (iii) those who experienced severe ongoing symptoms [“Ongoing (Severe) group,” *C* + + ; *n* = 66]. Those who were still at their first 3 weeks of COVID-19 infection (*n* = 17) or those who reported “it is too soon” to comment on their ongoing symptoms (*n* = 3) were not included in the following analyses. Participants in all groups ranged between 3 and 31 + weeks since symptom-onset, and a majority (81.5%) of those with ongoing symptoms reporting after more than 6 months since infection.

**FIGURE 3:**
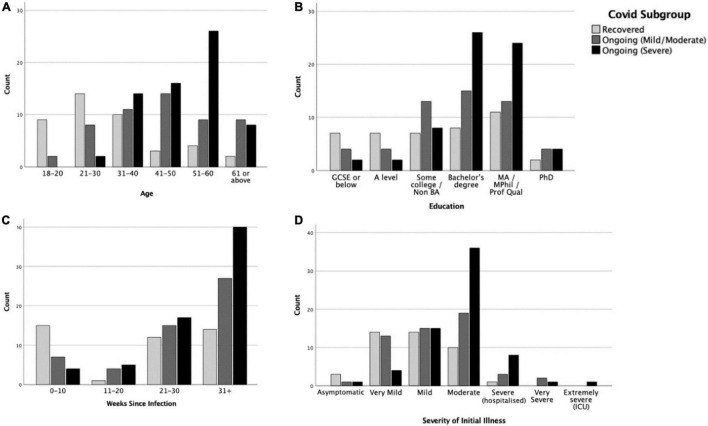
Distributions of **(A)** age, **(B)** education level, **(C)** weeks since infection, and **(D)** severity of initial illness in Recovered, Ongoing (Mild/Moderate) and Ongoing (Severe) subgroups.

Figure 3 shows the distribution of demographic variables across the COVID-19 duration subgroups (further details available in Supplementary Table 3). In each, more than half of the participants were from the United Kingdom (54.8–92.4%) and were of White (Northern European) ethnicity (69–93.9%). Pearson’s chi-square tests suggested that age [χ*^2^*(10) = 53.41, *p* < 0.001, *V* = 0.407] and education level [χ*^2^*(10) = 20.03, *p* = 0.029, *V* = 0.249], but not sex, significantly differed between subgroups. In terms of age, the *R* subgroup tended to fall more in the younger age ranges (see Figure 3A). In terms of education level, the *R* subgroup tended to have lower education level (GCSE or below and A level), but the *C* + + (Severe) subgroup clustered more in higher education level (bachelor’s degree) (see Figure 3B). The subgroups also differed in the time elapsed since infection at the time of completing the study [χ*^2^*(6) = 19.64, *p* = 0.003, *V* = 0.247]. The *R* subgroup were more likely to be in their first 10 weeks of infection, while the *C* + + (Severe) subgroup were more likely to be at their 31 weeks or above (Figure 3C).

A multinomial logistic regression indicated that only age, but not sex or education, was significantly associated with COVID-19 progression [χ*^2^*(10) = 43.6, *p* < 0.001]. People in the age ranges of 18–20 and 21–30 years were more likely to recover from COVID-19 than to progress into mild/moderate (*p*s = 0.02–0.03) or severe (*p* = 0.002) ongoing symptoms.

We examined whether medical history and health behaviors were different between COVID-19 duration subgroups. Table 2 shows the descriptive statistics of these factors in *R*, *C* +, and *C* + + subgroups for medical history and pre-pandemic health behaviors. None of the listed health conditions significantly differed between subgroups (against Sidak α = 0.003). There were, however, significant group differences (Sidak α = 0.008) in fruit and vegetables consumption [*H*(2) = 15.92, *p* < 0.001] and fatty food consumption [*H*(2) = 36.54, *p* < 0.001]. Both ongoing symptom subgroups ate more fruit and vegetables (*C* + + : *U* = 810, *p* < 0.001; *C* + : *U* = 808, *p* = 0.016) and less fatty food (*C* + : *U* = 773.5, *p* = 0.005; *C* + + : *U* = 552.5, *p* < 0.001) than the *R* subgroup. The *C* + (Mild/Moderate) subgroup also consumed more fatty food than the *C* + + (Severe) subgroup (*U* = 1142, *p* < 0.001). The subgroups did not significantly differ in BMI [*F*_(2, 157)_ = 0.085, *p* = 0.919].

**TABLE 2:** Distribution of medical history and health behaviors (1 = Never–6 = Several times daily; higher scores indicating higher frequency) in COVID subgroups: Recovered (R), Ongoing (Mild/Moderate) (C+) and Ongoing (Severe) (C++).

	Recovered (*R*) (*n* = 42)	Ongoing (Mild/Moderate) (*C* +) (*n* = 53)	Ongoing (Severe) (*C* + +) (*n* = 66)	

Medical history: Frequency (%)				Chi-square tests
Asthma	6 (14.3%)	10 (18.9%)	21 (31.8%)	n.s.
Depression	9 (21.4%)	12 (22.6%)	9 (13.6%)	n.s.
Other mental health disorder	12 (28.6%)	9 (17%)	4 (6.1%)	χ*^2^*(2) = 10.04, *p* = 0.007, *V* = 0.250
Obesity	6 (14.3%)	8 (15.1%)	6 (9.1%)	n.s.
High blood pressure	3 (7.1%)	10 (18.9%)	6 (9.1%)	n.s.
History of migraines	4 (9.5%)	6 (11.3%)	7 (10.6%)	n.s.
Inflammatory/Autoimmune	4 (9.5%)	6 (11.3%)	8 (12.1%)	n.s.
Chronic fatigue syndrome/Myalgic encephalomyelitis (ME)	−	2 (3.8%)	5 (7.6%)	n.s.
Psychiatric/Neurodevelopmental disorder	2 (4.8%)	2 (3.8%)	3 (4.5%)	n.s.
Cardiovascular disease/Angina	−	3 (5.7%)	3 (4.5%)	n.s.
Diabetes (Type 2)	−	1 (1.9%)	1 (1.5%)	n.s.
Diabetes (Type 1)	−	−	−	n.s.
Cancer	−	−	2 (3%)	n.s.
A clotting disorder	1 (2.4%)	−	1 (1.5%)	n.s.
None of the above	15 (35.7%)	14 (26.4%)	24 (36.4%)	n.s.

**Health Behaviors: *Mean (SD)***				**Kruskal-Wallis *H*-tests/Mann-Whitney *U*-tests**

Diet: Fruit and vegetables	4.52 (1.29)	5.15 (0.95)	5.41 (0.93)	*H*(2) = 15.92, *p* < 0.001* *C* + + > *R*: *U* = 810, *p* < 0.001* *C* + > *R*: *U* = 808, *p* = 0.016*
Diet: Sugary food	3.71 (1.2)	3.34 (0.9)	3.24 (1.05)	n.s.
Diet: Fatty food	3.6 (0.94)	3.11 (0.8)	2.58 (0.63)	*H*(2) = 36.54, *p* < 0.001* *R* > *C* + :*U* = 773.5, *p* = 0.005* *R* > *C* + + : *U* = 552.5, *p* < 0.001* *C* + > *C* + + : *U* = 1,142, *p* < 0.001*
Physical activity	3.31 (1.18)	4.04 (1.16)	3.85 (1.51)	*H*(2) = 9.03, *p* = 0.011 *C* + + > *R*: *U* = 1,027, *p* = 0.02 *C* + > *R*: *U* = 722.5, *p* = 0.003
Alcohol	2.81 (0.97)	2.68 (1.11)	2.47 (1.01)	n.s.
Smoking	1.48 (1.17)	1.57 (1.47)	1.15 (0.86)	*H*(2) = 8.42, *p* = 0.015 *C* + > *C* + + : *U* = 1,542, *p* = 0.021

* denotes p-values below Sidak-correct alpha (i.e., non-null).

After controlling for sex, age, education, and country, a forward stepwise multinomial logistic regression indicated that no medical history variables were associated with COVID-19 progression, however, health behaviors including fatty food consumption [χ*^2^*(2) = 23.25, *p* < 0.001], physical activity [χ*^2^*(2) = 10.31, *p* = 0.006], and alcohol consumption [χ*^2^*(2) = 8.18, *p* = 0.017] were all significantly associated with COVID-19 progression. In our sample, people consuming more fatty food had a higher chance of having recovered from COVID-19 (*p* < 0.001) or having developed mild/moderate ongoing symptoms (*p* < 0.001) than progressing into severe ongoing symptoms. Higher levels of physical activity were associated with reduced chance of recovery relative to progression onto mild/moderate (*p* = 0.002) or severe ongoing symptoms (*p* = 0.034). Those drinking alcohol more frequently were more likely to recover from COVID-19 than to develop severe ongoing symptoms (*p* = 0.007).

#### Severity of Initial Illness

The severity of illness in the first 3 weeks of infection was associated with subsequent symptom longevity. Multinomial logistic regression showed that severity of initial illness was significantly associated with COVID-19 progression [χ*^2^*(2) = 24.44, *p* < 0.001], with higher initial severity associated with more severe subsequent ongoing symptoms (*p*s < 0.001–0.02). This effect was maintained after controlling for sex, age, education, and country [χ*^2^*(2) = 12.28, *p* = 0.002; *C* + + > *C* + : *p* = 0.048; *C* + + > *R*: *p* = 0.001]. Those with severe ongoing symptoms experienced more severe initial illness than those whose ongoing symptoms were mild/moderate (*U* = 1,258, *p* = 0.005, Figure 3D) and those who were fully recovered (*U* = 658.5, *p* < 0.001). The severity difference between the *C* + (Mild/Moderate) subgroup and the *R* subgroup was also significant (*U* = 842, *p* = 0.034).

Supplementary Table 4 shows the relative frequencies of particular diagnoses received during the initial illness. Of the 109 participants who sought medical assistance, the most common diagnoses received were hypoxia (14.7%), blood clots (5.5%), and inflammation (4.6%).

#### Symptoms During Initial Illness

**FIGURE 4:**
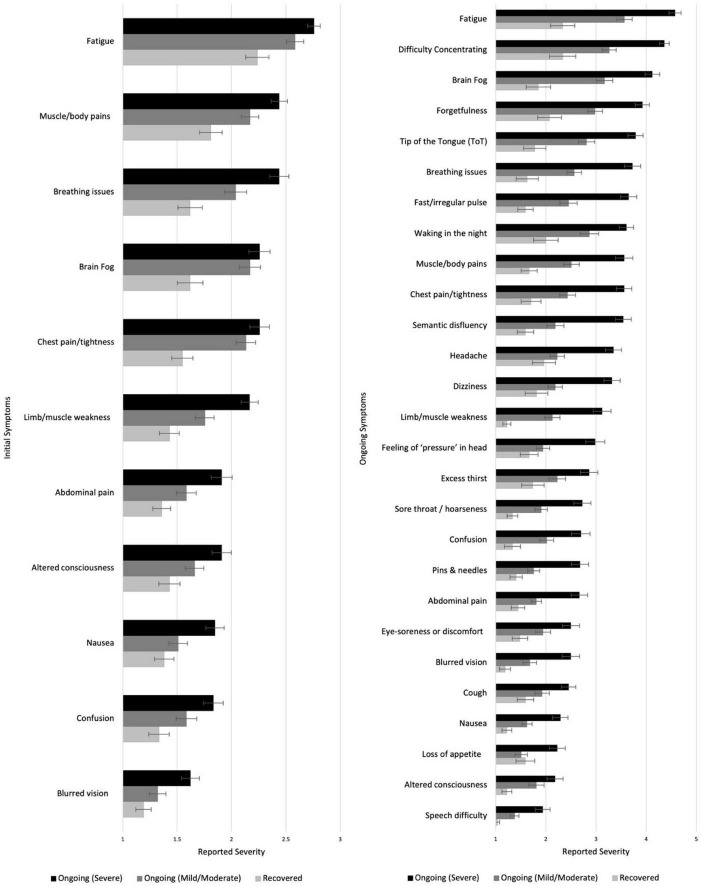
Severity of different symptoms during the initial **(left)** and ongoing **(right)** illness among those who recovered or had ongoing mild or severe illness. Higher scores indicate higher severity.

Symptoms that appeared in less than 10% of participants were excluded. Kruskal-Wallis *H*-tests (Sidak α = 0.001) showed significant duration-group differences in 11/33 symptoms in terms of the severity experienced (see Figure 4, more information in Supplementary Table 5). In *post hoc* analysis (Sidak α = 0.017), muscle/body pains, breathing issues and limb weakness showed gradation, with the *C* + + (Severe) subgroup having experienced the most severe symptoms, followed by the *C* + (Mild/Moderate) subgroup, and the *R* subgroup experiencing the least (*p* ranges < 0.001–0.012). Some symptoms did not show gradation with severity of ongoing symptoms, but were reliably higher in those with ongoing symptoms. Both the ongoing symptoms subgroups reported more severe symptoms of fatigue, brain fog and chest pain/tightness during the initial illness than those that recovered (*p*s < / = 0.001) but did not differ from one another. Those with severe ongoing symptoms experienced more severe nausea and blurred vision than those with mild/moderate or who recovered (*p* ranges < 0.001–0.009). Finally, the *C* + + (Severe) subgroup experienced more abdominal pain, altered consciousness and confusion during the initial illness than the *R* subgroup (*p*s < / = 0.001).

After controlling for sex, age, education, and country, a forward stepwise multinomial logistic regression suggested that six initial symptoms were significantly associated with COVID-19 progression. These were: limb weakness [χ*^2^*(2) = 25.92, *p* < 0.001], brain fog [χ*^2^*(2) = 13.82, *p* = 0.001], chest pain or tightness [χ*^2^*(2) = 10.81, *p* = 0.005], dizziness [χ*^2^*(2) = 7.82, *p* = 0.02], cough [χ*^2^*(2) = 7.74, *p* = 0.021], and breathing difficulties [χ*^2^*(2) = 6.98, *p* = 0.031]. People initially experiencing more severe limb weakness were more likely to experience severe ongoing symptoms than to recover (*p* < 0.001) or develop mild/moderate ongoing symptoms (*p* < 0.001). More severe initial breathing issues (*p* = 0.014) and dizziness (*p* = 0.037) were associated with greater likelihood of severe than mild/moderate ongoing symptoms, but people with more severe initial dizziness (*p* = 0.02) and cough (*p* = 0.009) were more likely to recover rather than to develop mild/moderate ongoing symptoms. More severe initial brain fog and chest pain/tightness were associated with more progression into mild/moderate than either severe ongoing symptoms (brain fog: *p* = 0.029; chest pain: *p* = 0.026) or recovery (brain fog: *p* = 0.001; chest pain: *p* = 0.007).

#### Symptoms During Ongoing Illness

Excluding those who reported being totally asymptomatic throughout or feeling completely better very quickly after initial illness (who did not report on ongoing symptoms, *n* = 15), the COVID subgroups were asked to report on their ongoing experience of a list of 52 symptoms. Symptoms that appeared in less than 10% of participants were excluded. The duration-groups differed significantly in 27/47 symptoms (Sidak α = 0.001; see Figure 4 and Supplementary Table 6). *Post hoc* tests (Sidak α = 0.017) showed that the *C* + + (Severe) subgroup reported higher levels of severity than the *R* subgroup in all 27 symptoms (*p*s < 0.001–0.017) and then the *C* + (Mild/Moderate) subgroup in all except two (altered consciousness and eye-soreness; *p*s < 0.001–0.017). The *C* + (Mild/Moderate) subgroup also reported experiencing higher severity in 16 symptoms (including fatigue, difficulty concentrating, brain fog, and forgetfulness) than the *R* subgroup (*p*s < 0.001–0.016; see Figure 4 and Supplementary Table 6; see also Supplementary Table 7 for similar analysis of current symptoms).

### Symptoms in Those With Confirmed or Suspected COVID-19 vs. “Other” Illnesses

As much of our sample experienced infection early in the pandemic before widespread testing was available, not all cases included in our COVID group were confirmed by a polymerase chain reaction (PCR) test (infection statuses: “Confirmed” COVID, “Unconfirmed” COVID). Meanwhile, a significant minority of participants had an illness during the pandemic period that they did *not* think was COVID-19 (infection status: “Unknown”) (see Figure 1). We compared symptom prevalence across these three groups (Unknown, *n* = 55; Unconfirmed, *n* = 96; Confirmed, *n* = 65) for both the initial 3 weeks of illness, and the time since then. Those who were still at their first 3 weeks of COVID-19 infection (*n* = 17) and who reported “it is too soon” to comment on their ongoing symptoms (*n* = 3) were not included in this analysis.

The groups significantly differed in 14 out of 31 symptoms during the initial illness (Sidak α = 0.0016; Supplementary Table 8). Both Confirmed and Unconfirmed groups reported higher severity than the Unknown group on 13 symptoms (including fatigue, muscle/body pains and loss of smell/taste; *p* ranges < 0.001–0.014; Sidak α = 0.017). Additionally, the Unconfirmed group reported more severe blurred vision than the Unknown group (*p* < 0.001), and the Unknown group reported more severe sore throat/hoarseness than the Confirmed group (*p* < 0.001). As for the differences within those with COVID-19, the Confirmed group experienced greater loss of smell/taste than the Unconfirmed group (*p* = 0.002), while the Unconfirmed group reported higher levels of breathing issues, chest pain/tightness, sore throat/hoarseness, and blurred vision than the Confirmed group (*p*s = 0.004–0.015).

**FIGURE 5:**
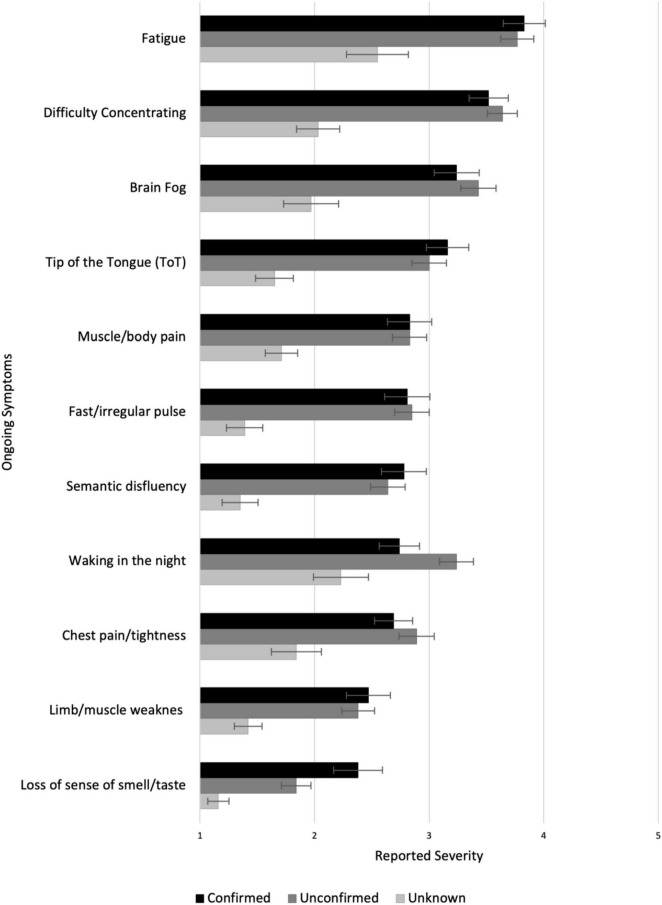
Experience of ongoing symptoms in Unknown, Unconfirmed COVID, and Confirmed COVID groups.

Of these participants, 177 (Unknown group: *n* = 31; Unconfirmed group: *n* = 88; Confirmed group: *n* = 58) reported experiencing ongoing symptoms after the 3 weeks of illness. Significant group differences were found in 11/47 ongoing symptoms (Sidak α = 001; see Figure 5 and Supplementary Table 9). *Post hoc* tests (Sidak α = 0.017) showed that, compared with the Unknown group, both the Confirmed and Unconfirmed groups reported higher levels of fatigue, difficulty concentrating, brain fog, tip-of-the-tongue (ToT) problems, muscle/body pains, fast/irregular pulse, semantic disfluency, chest pain/tightness, limb weakness, and loss of smell/taste (*p*s < / = 0.001). The Unconfirmed group also experienced higher level of night waking (*p* = 0.001) than the Unknown group. There were no significant differences in ongoing symptoms between the Confirmed and the Unconfirmed groups.

### Characterizing Symptom Profiles

While data on individual symptoms are useful in identifying highly specific predictors, these are too numerous for more systematic analysis, which require data-reduction. A stated aim of this study was to identify symptom profiles that may be informative as to underlying pathology.

#### Initial Symptom Factors

To group the initial symptoms, we included 34 symptoms in the PCA after excluding paralysis and seizures (experienced by less than 10% of the participants). A total of 164 participants reported on their symptoms during the first 3 weeks of illness (the factor analysis coded here as 1 = *Very severe*, 3 = *Not at all*). The Kaiser-Meyer-Olkin (KMO) test (value 0.861) and Bartlett’s test of sphericity [χ*^2^*(528) = 2,250, *p* < 0.001] showed the data were suitable for factor analysis. We employed the varimax rotation. Initially, nine factors were obtained with eigenvalue > 1.0, which was reduced to five via Cattell’s Scree test (Kline, 2013). Assessments were conducted of 4, 5, and 6 factor solutions for interpretability and robustness. The ratio of rotated eigenvalue to unrotated eigenvalue was higher for the 5-factor solution than for the 4- or 6-factor solutions, and this structure was also the most interpretable. We thus proceeded with a 5-factor solution, which explained 50.59% of item variance with last rotated eigenvalue of 1.998.

We labeled the new components as “F1: Neurological/Psychiatric,” “F2: Fatigue/Mixed,” “F3: Gastrointestinal,” “F4: Respiratory/Infectious,” and “F5: Dermatological” (see Table 3 for factor loadings). We computed the factor scores using the regression method (see Supplementary Table 10 for factor scores).

**TABLE 3:** Factors and loadings from the “Initial Symptoms” PCA.

	Component				
Symptom	F1 Neurological/Psychiatric	F2 Fatigue/Mixed	F3 Gastrointestinal	F4 Respiratory/Infectious	F5 Dermatological
Disorientation	**0.763**				
Delirium	**0.688**				
Visual disturbances	**0.639**				
Confusion	**0.630**	0.431			
Altered consciousness	**0.617**	0.364			
Speech difficulty	**0.583**				
Blurred vision	**0.518**	0.374			
Hallucinations	**0.502**				
Drowsiness	**0.453**	0.362			
Anxiety	**0.416**				
Numbness	**0.367**	0.346			
Fatigue		**0.753**			
Chest pain/tightness		**0.631**		0.313	
Muscle/body pains		**0.585**			
Headache		**0.543**	0.368		
Limb weakness		**0.541**			0.301
Dizziness	0.395	**0.530**			
Brain fog	0.466	**0.523**			
Eye-soreness	0.325	**0.511**			
Diarrhea			**0.738**		
Nausea		0.307	**0.707**		
Vomiting			**0.696**		
Abdominal pain		0.315	**0.649**		
Acid reflux		0.323	**0.403**		
Sore throat			**0.338**		
Fever				**0.717**	
Cough				**0.609**	
Breathing issues		0.479		**0.592**	
Loss of appetite				**0.526**	
Loss of smell/taste				**0.361**	
Rash					**0.785**
Itchy welts					**0.782**
Foot sores			0.426		**0.586**
Face/lips swelling				0.367	**0.490**

*The bold indicates items loading above 0.5; non bold numbers are those loading above 0.3.*

**FIGURE 6:**
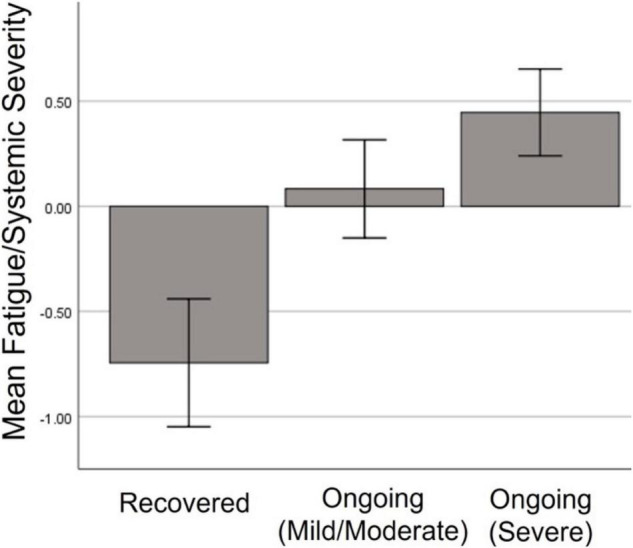
Severity of Fatigue/Mixed symptom factor during initial illness among those who went on to full recover, or have ongoing mild or severe symptoms.

People who went on to experience ongoing symptoms showed higher factor scores in the Fatigue/Mixed symptom factor during the initial illness [*F*_(2, 158)_ = 23.577, *p* < 0.001], but did not differ in any other initial symptom factor. Pairwise analysis revealed that those who recovered were significantly less likely to experience Fatigue/Mixed symptoms than those with mild/moderate (*p* < 0.001) or severe (*p* < 0.001) ongoing symptoms (Figure 6).

#### Ongoing Symptom Factors

We performed a second PCA using the symptoms experienced since the initial phase (after the first 3 weeks), including 45 symptoms. Paralysis and seizures were excluded (experienced by less than 10% of the participants). A total of 149 participants reported on their symptoms over the time since the first 3 weeks of illness (the factor analysis coded here as 1 = *Very severe and often*, 5 = *Not at all*). The KMO test (value 0.871) and Bartlett’s test of sphericity [χ*^2^*(861) = 3,302, *p* < 0.001] showed suitability for factor analysis. We employed the varimax rotation. PCA showed 11 components with eigenvalues > 1.0, and this was reduced to 6 via inspection of the eigenvalue gradient (scree plot). The ratio of rotated eigenvalue to unrotated eigenvalue was higher for the 7-factor solution, followed by the 6-factor. The 6- and 7-factor solutions were differentiated by subdivision of the second factor, reducing the degree of cross-loading. However, the 7-factor solution was less interpretable and less robust to removal to cross-loaders (the presence of which can be accepted from a pathology perspective, given that multiple mechanisms can produce the same symptom). As such, we proceeded with the 6-factor solution, which explained 54.17% of item variance and had a last rotated eigenvalue of 2.227.

We labeled the new components as “F1: Neurological,” “F2: Gastrointestinal/Autoimmune,” “F3: Cardiopulmonary/Fatigue,” “F4: Dermatological/Fever,” “F5: Appetite Loss,” and “F6: Mood” (see Table 4 for factor loadings). We computed the factor scores using the regression method (see Supplementary Table 11 for factor scores).

**TABLE 4:** Factors and loadings from the exploratory factor analysis of ongoing “since then” symptoms PCA.

	Component					
Symptom	F1 Neurological	F2 Gastrointestinal/Autoimmune	F3 Cardiopulmonary/Fatigue	F4 Dermatological/Fever	F5 Appetite Loss	F6 Mood
Disorientation	**0.695**					0.323
Confusion	**0.651**					
Delirium	**0.639**					
Speech difficulty	**0.619**					
Altered consciousness	**0.607**					0.316
Visual disturbances	**0.604**			0.386		
Hallucinations	**0.576**			0.386		0.301
Pins & needles	**0.561**	0.399				
Numbness	**0.559**					
Blurred vision	**0.531**	0.369		0.348		
Head pressure	**0.501**	0.428				
Drowsiness	**0.490**					
Hot flushes		**0.624**		0.306		
Nausea		**0.608**				
Diarrhea		**0.591**				
Abdominal pain		**0.576**		0.309		
Headache		**0.565**	0.301			
Muscle/body pains		**0.563**	0.524			
Eye-soreness	0.305	**0.488**			0.342	
Dizziness	0.435	**0.477**	0.373			
Weight gain		**0.471**			−0.396	
Acid reflux		**0.456**				
Incontinence		**0.393**				
Breathing issues			**0.793**			
Chest pain/tightness			**0.727**			
Fatigue		0.391	**0.619**			
Cough			**0.580**	0.330		
Fast/irregular pulse		0.430	**0.553**			
Night waking			**0.536**			
Limb weakness	0.428	0.457	**0.466**			
Difficulty sleeping			**0.457**		0.356	0.345
Sore throat	0.308	0.324	**0.388**			
Face/lips swelling				**0.678**		
Foot sores				**0.646**		
Itchy welts				**0.562**		
Rash		0.303		**0.549**		
Fever				**0.461**		
Loss of smell/taste				**0.421**		
Excess thirst		0.305	0.316	**0.390**		
Vomiting		0.321		**0.385**		
Weight loss					**0.752**	
Loss of appetite					**0.637**	
Depression						**0.715**
Anxiety	0.316					**0.683**
Vivid dreams		0.337				**0.428**

*The bold indicates items loading above 0.5; non bold numbers are those loading above 0.3.*

In order for cognitive symptoms [brain fog, forgetfulness, tip-of-the-tongue (ToT) problems, semantic disfluency and difficulty concentrating] to be used as a dependent variable, these were isolated and a PCA run separately. A single component emerged, with all the cognitive symptoms loading homogeneously highly (see Supplementary Table 12). The KMO test (value 0.886) and Bartlett’s test of sphericity [χ*^2^*(10) = 564, *p* < 0.001] indicated suitability for factor analysis, and the single 5-item factor explained 76.86% of variance.

#### Current Symptoms

The current symptoms assessed were the same as the ongoing symptoms, but rated dichotomously as either currently present or absent. To estimate the degree to which current symptoms aligned with the factors established for the ongoing period, we generated a quasi-continuously distributed variable according to how many of the high loading (> / = 0.5) items from the ongoing factors were recorded as present currently. Using this *sum scores by factor* method (Tabachnick et al., 2007; Hair, 2009), each score was subsequently divided by the number of items in that factor producing quasi “factor scores” that were comparable and indicative of “degree of alignment” of current symptoms to established factors.

To assess the stability and specificity of symptom profiles between these periods, serial correlations were conducted for corresponding and non-corresponding factors. Correlations of the same factor across time points were materially higher (> 0.2) from the next highest correlation among the 5 non-corresponding factors, with Williams tests (Steiger, 1980) giving the narrowest gap at *p* = 0.003 (Neurological: *r* = 0.676, *t* = 5.712; Gastrointestinal/Autoimmune: *r* = 0.531, *t* = 3.778; Cardiopulmonary/Fatigue: *r* = 0.678, *t* = 7.272; Dermatological/Fever: *r* = 0.523, *t* = 3.364; Appetite Loss: *r* = 0.591, *t* = 5.017; Mood: *r* = 0.490, *t* = 4.803). This consistency suggests that while particular symptoms may fluctuate, the profile of symptoms—once grouped into an adequately supported factor—is moderately stable for individuals, and can be relatively well represented by a “snapshot” of current symptoms. For completeness, an additional factor analysis was conducted on the current symptoms, which are reported in Supplementary Table 13.

**FIGURE 7:**
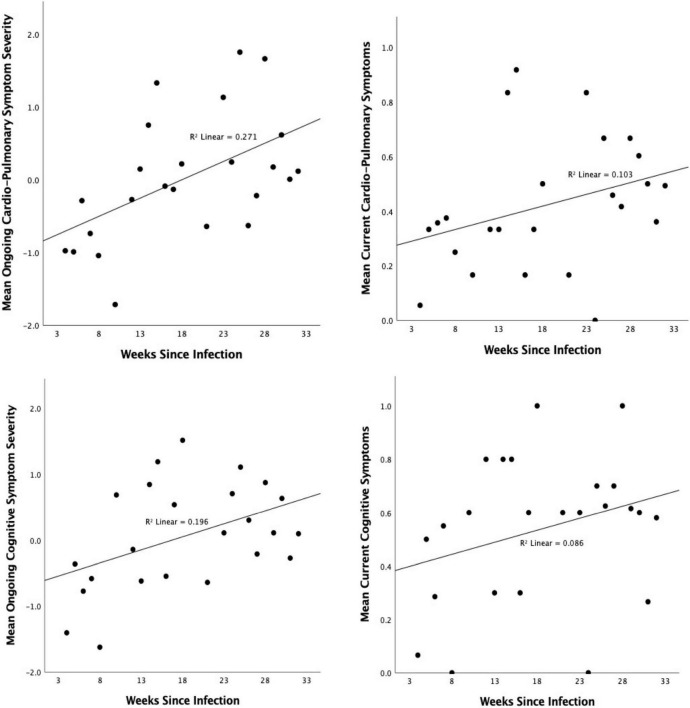
Association between number of weeks since infection and severity of **(top)** Cardiopulmonary/Fatigue Symptoms and **(bottom)** cognitive symptoms in the entire period since the initial infection **(left)** and the past 1–2 days **(right)**. Higher scores indicate higher symptom severity.

One symptom factor showed change over time since infection, suggesting higher severity in those who had been ill for longer: Number of weeks since infection (positive test/first symptoms) was positive correlated with severity of ongoing severity of Cardiopulmonary/Fatigue symptoms [*r*(147) = 0.271, *p* < 0.001; Figure 7] and, to a weaker extent, current alignment with the same factor [*r*(147) = 0.206, *p* = 0.012], however, only the former association survived correction for multiple comparisons (Sidak α = 0.0085).

#### Cognitive Symptoms

Within those currently experiencing symptoms (*n* = 126), 77.8% reported difficulty concentrating, 69% reported brain fog, 67.5% reported forgetfulness, 59.5% reported tip-of-the-tongue (ToT) word finding problems and 43.7% reported semantic disfluency (saying or typing the wrong word).

**FIGURE 8:**
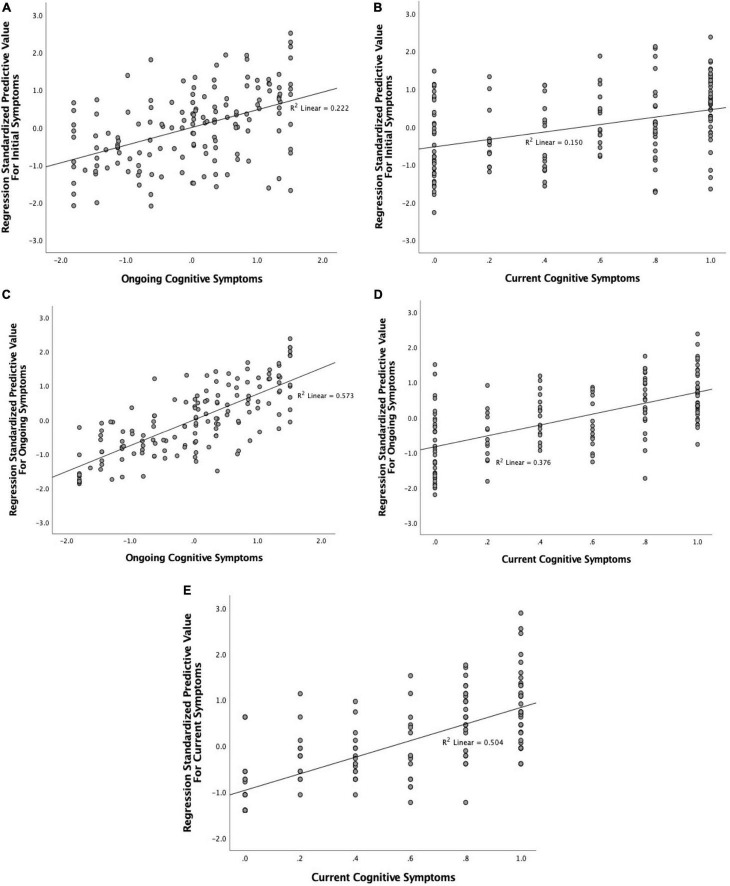
Association between combined regression model predicted value for **(A)** initial symptom factors and ongoing cognitive symptoms; **(B)** initial symptom factors and current cognitive symptoms; **(C)** ongoing symptom factors and ongoing cognitive symptoms; **(D)** ongoing symptom factors and current cognitive symptoms; and **(E)** current symptom factors and current cognitive symptoms.

Symptoms experienced during the initial illness significantly predicted both ongoing and current cognitive symptoms (Figure 8). A linear regression with backward elimination found that the best model contained the Neurological/Psychiatric, Fatigue/Mixed, Gastrointestinal, and Respiratory/Infectious symptom factors and explained 20% of variance (*R_*adj*_^2^* = 0.2, *p* < 0.001). Table 5 shows that the Fatigue/Mixed symptoms factor (η′*_*p*_*^2^ = 0.129) was the better predictor followed by the Neurological/Psychiatric symptom factor (η′*_*p*_*^2^ = 0.092). For current cognitive symptoms, the best model contained both the Neurological/Psychiatric and Fatigue/Mixed symptom factors, together explaining 13.9% of variance (*p* < 0.001). Of the two, the Fatigue/Mixed factor was the better predictor (η′*_*p*_*^2^ = 0.110). No interactions between factors contributed significantly and were thus not included in the final models.

**TABLE 5:** Regression models predicting variation in the cognitive symptom factor (ongoing and current) from non-cognitive symptom factors (initial, ongoing, and current).

	*R_*adj*_^2^*	Effect size (η′*_*p*_*^2^) of Independent Variable						Interactions

IV: Initial symptoms								

		Neurological/Psychiatric	Fatigue/Mixed	Gastrointestinal	Respiratory/Infectious	Dermatological		
Ongoing Cognitive Symptoms	0.2 *p* < 0.001	0.092	0.129	0.029	0.029	n.s.		
Current Cognitive Symptoms	0.139 *p* < 0.001	0.057	0.110	n.s.	n.s.	n.s.		

**IV: Ongoing symptoms**								

		**Neurological**	**Gastrointestinal/Autoimmune**	**Cardiopulmonary/Fatigue**	**Dematological/Fever**	**Appetite loss**	**Mood**	**GI/AI × Card-Pul**

Ongoing Cognitive Symptoms	0.558 *p* < 0.001	0.236	0.309	0.325	n.s.	0.056	0.043	
Current Cognitive Symptoms	0.36 *p* < 0.001	0.118	0.115	0.208	n.s.	n.s.	n.s.	0.038
**IV: Current symptoms**								
Current Cognitive Symptoms	0.494 *p* < 0.001	0.074	n.s.	0.306	n.s.	0.021	n.s.	

*Only partial eta squared (η′_p_^2^) effect size is given here, as beta coefficients are not meaningful for already standardized variables.*

A similar, but much stronger, pattern emerged when considering the predictive value of ongoing (non-cognitive) symptoms (Figure 8). Using backward elimination to factors with significance (*p* < 0.05), all factors except Dermatological/Fever remained in the model, which explained over 55% of variance (*R_*adj*_^2^* = 0.558, *p* < 0.001). The effect size (η′*_*p*_*^2^) for each factor is given in Table 5. The Gastrointestinal/Autoimmune and Cardiopulmonary/Fatigue factors were the biggest contributors to the model. Indeed, in an extreme elimination model in which contributing factors were limited to two or fewer, these two factors alone explained 38% of variance retaining strong significance (*p* < 0.001). No interactions between factors contributed significantly and were thus not included in the final models. Ongoing symptoms also predicted current cognitive symptoms. The best model explained 36% of the variance (*p* < 0.001) and included the Neurological, Gastrointestinal/Autoimmune and Cardiopulmonary/Fatigue factors and an interaction between the Gastrointestinal/Autoimmune and Cardiopulmonary/Fatigue factors. Of these, Cardiopulmonary/Fatigue symptoms were the strongest predictor (η′*_*p*_*^2^ = 0.208), with Neurological (η′*_*p*_*^2^ = 0.118) and Gastrointestinal/Autoimmune (η′*_*p*_*^2^ = 0.115) being relatively equal.

Current symptom factors also strongly predicted current cognitive symptoms (Figure 8). The backward elimination model left three contributing factors: Neurological, Cardiopulmonary/Fatigue and Appetite Loss. Together these explained around 50% of variance (*R_*adj*_^2^* = 0.494). Of these, Cardiopulmonary/Fatigue was the stronger predictor (η′*_*p*_*^2^ = 0.306). Indeed, when the model was limited to just this factor, this model still explained 43% of the variance.

There was a significant association between degree of cognitive symptoms and duration of illness. Those who had been ill for longer were more likely to report having had cognitive symptoms throughout the ongoing illness [*r*(147) = 0.262, *p* = 0.001] and to be experiencing them at the time of test [*r*(147) = 0.179, *p* = 0.03] (Figure 7).

### Experiences and Impact of Long COVID

Here we limited analysis to all those who reported some degree or period of ongoing symptoms following COVID-19 [i.e., excluding those who reported being totally asymptomatic throughout or feeling completely better very quickly after initial illness (*n* = 15)]. Of the remaining 146 participants, 108 (74%) self-identified as experiencing or having experienced “Long COVID.”

We examined the impact and experiences of ongoing illness (Table 6). In most cases, the nature and degree of negative experience of ongoing symptoms scaled with perceived severity. The change in symptoms over time differed between severity subgroups [χ*^2^*(6) = 37.52, *p* < 0.001, *V* = 0.367]. The *C* + + (Severe) subgroup were more likely to report that symptoms were consistent over time, while those with mild/moderate ongoing symptoms were more likely to report improvement in symptoms. As might be expected, the *R* subgroup were alone in reporting complete resolution of symptoms after recovery from the initial illness (Supplementary Table 14).

**TABLE 6:** Experiences and impact of Long COVID in different ongoing symptom severity groups.

	Now Recovered (*R*) (*n* = 27**)	Ongoing (Mild/Moderate) (*C* +) (*n* = 53)	Ongoing (Severe) (*C* + +) (*n* = 66)	Chi-square tests
**Identify as experiencing “Long COVID”**				χ*^2^*(4) = 85.75, *p* < 0.001, *V* = 0.542
Yes	3 (11.1%)	43 (81.1%)	62 (93.9%)	
No	16 (59.3%)	2 (3.8%)	−	
Other	8 (29.6%)	8 (15.1%)	4 (6.1%)	
**Change of symptoms after initial illness**				χ*^2^*(6) = 37.52, *p* < 0.001, *V* = 0.367
No ongoing symptoms after initial recovery	5 (18.5%)	−	−	
Different symptoms at different times	8 (29.6%)	28 (52.8%)	39 (59.1%)	
Improvement in symptoms over time	5 (18.5%)	18 (34%)	9 (13.6%)	
Symptoms have been very consistent	3 (11.1%)	7 (13.2%)	17 (25.8%)	
I don’t know/N/A	6 (22.2%)	−	1 (1.5%)	
**Cycle of symptoms after initial illness**				n.s.
Cycle every few days	3 (11.1%)	11 (20.8%)	14 (21.2%)	
Cycle every few weeks	3 (11.1%)	13 (24.5%)	19 (28.8%)	
Cycle monthly	2 (7.4%)	7 (13.2%)	9 (13.6%)	
No cycling	12 (44.4%)	19 (35.8%)	23 (34.8%)	
N/A	7 (25.9%)	3 (5.7%)	1 (1.5%)	
**Impact of Long COVID**				
Long period unable to work (due to illness)	2 (7.4%)	15 (28.3%)	50 (75.8%)	χ*^2^*(2) = 46.42, *p* < 0.001, *V* = 0.564*
Difficulty coping day-to-day activities	6 (22.2%)	28 (52.8%)	48 (72.7%)	χ*^2^*(2) = 20.23, *p* < 0.001, *V* = 0.372*
Difficulty getting medical professionals to take symptoms seriously	1 (3.7%)	21 (39.6%)	38 (57.6%)	χ*^2^*(2) = 23.05, *p* < 0.001, *V* = 0.397*
Lost job due to illness	1 (3.7%)	9 (17%)	32 (48.5%)	χ*^2^*(2) = 24.39, *p* < 0.001, *V* = 0.409*
Feeling that you have experienced a trauma	4 (14.8%)	21 (39.6%)	31 (47%)	χ*^2^*(2) = 8.44, *p* = 0.015, *V* = 0.240
Financial difficulty (as a result of illness)	1 (3.7%)	7 (13.2%)	14 (21.2%)	n.s.
None of the above	18 (66.7%)	9 (17%)	1 (1.5%)	χ*^2^*(2) = 52.73, *p* < 0.001, *V* = 0.601*

*Denotes p-values below Sidak-correct alpha at 0.007 for the impact of Long COVID.

**Excluding a small portion of participants who reported asymptomatic or feeling completely better very quickly from the Recovered subgroup (n = 15).

Long COVID has significant impact on individuals’ lives. Over 54.6% of those with ongoing symptoms had experienced long periods unable to work and 34.5% had lost their job due to illness, 63.9% reported difficulty coping with day-to-day activities, 49.6% had had difficulty getting medical professionals to take their symptoms seriously, and 43.7% felt that they had experienced a trauma, while 17.6% had experienced financial difficulty as a result of illness. These impacts scaled with symptom severity. Those with severe ongoing symptoms were more likely to report being unable to work for a long period due to illness [χ*^2^*(2) = 46.42, *p* < 0.001, *V* = 0.564], having difficulty coping with day-to-day requirements [χ*^2^*(2) = 20.23, *p* < 0.001, *V* = 0.372], having difficulty getting medical professionals to take their symptoms seriously [χ*^2^*(2) = 23.05, *p* < 0.001, *V* = 0.397], and losing their job due to illness [χ*^2^*(2) = 24.39, *p* < 0.001, *V* = 0.409]. In contrast, the *R* subgroup tended to report experiencing none of the above [χ*^2^*(2) = 52.73, *p* < 0.001, *V* = 0.601].

We further compared job-loss with the No COVID group (*n* = 185). Those with ongoing symptoms were more likely to have lost their job than those who had not experienced COVID-19 [χ*^2^*(1) = 26.74, *p* < 0.001, *V* = 0.297]. The most common reason for job-loss among those with ongoing symptoms was illness [χ*^2^*(1) = 56.85, *p* < 0.001, *V* = 0.432], while the most common reason in the No COVID group was economy [χ*^2^*(1) = 7.67, *p* = 0.006, *V* = 0.159].

## Discussion

### Nature of Illness and Symptom Profiles

Here we report the initial findings from a cross-sectional/longitudinal study investigating cognition post-COVID-19. One aim of this first publication was to characterize the “COVID and Cognition Study” (COVCOG) sample. Within the COVID group, we recruited specifically to get good representation of those who were experiencing or had experienced ongoing symptoms. Indeed, 74% identified with the term “Long COVID.” Our final sample had a relatively even spread of those that had fully recovered at the time of test (42), or had mild/moderate (53) or severe (66) ongoing symptoms. Medical history did not differ between those experiencing ongoing symptoms and those who recovered. However, in terms of health behaviors, those with ongoing symptoms were in general “healthier,” being more likely to have previously been consuming less fatty food and more fruits and vegetables. This result is counterintuitive and may reflect insufficient controls for confounding demographic variables relating to socio-economic status. Nonetheless potential links between lifestyle and nutrition and COVID-19 recovery warrant further investigation.

The nature of the initial illness was found to have a significant impact on the likelihood and severity of ongoing symptoms. Despite this sample almost entirely comprised of non-hospitalized patients, those with more severe initial illness were more likely to have ongoing symptoms, and for those symptoms to be more severe. This suggests even in “community” cases, initial infection severity is a predictor of vulnerability to Long COVID. In an analysis of all symptoms experienced during the initial illness, there were several that were predictive of presence or severity of ongoing symptoms. In particular, individuals with severe ongoing symptoms were significantly more likely to have experienced limb weakness during the initial illness than those that recovered. However, some differences in severity ratings between ongoing subgroups were small despite being statistically significant, which warrant caution in interpreting the results.

We asked participants to retrospectively report on symptoms over three time periods: initial illness, ongoing illness, and currently experienced. Given the highly heterogenous nature of Long COVID, we used principal component analysis (PCA) with the aim to ascertain whether there may be different phenotypes of the condition within our sample—that is to say, that there may be certain types of symptoms that tend to (or not to) co-occur. For both the initial and ongoing illness, the symptom factors resemble those found in previous studies (e.g., Davis et al., 2021; Whitaker et al., 2021; Ziauddeen et al., 2021), with some quite coherent cardiopulmonary clusters, and other less specific “multisystem” profiles which may reflect more systemic issues such as inflammation, circulation, or endocrine function.

### Predictors of Cognitive Difficulties

A large proportion of our sample reported cognitive difficulties. We isolated the cognitive symptoms for the ongoing and current illness and computed a single factor including only these. Using this, we investigated which (non-cognitive) symptom factors during both the initial and ongoing illness explained significant variance in severity of cognitive symptoms.

Together, the Fatigue/Mixed, Neurological/Psychiatric, Gastrointestinal and Respiratory/Infectious symptom factors during the initial illness explained around 20% of variance in ongoing (“since then”) cognitive symptoms, and a similar model (containing only Neurological/Psychiatric and Fatigue/Mixed symptom factors) explained nearly 14% of variance in current cognitive symptoms. These findings strongly suggest that experience of neurological symptoms during the initial illness are significant predictors of self-reported cognitive impairment. While only one factor is named “Neurological” both this and the Fatigue/Mixed factor contain clear elements of neurological involvement. Indeed, headache, dizziness, and brain fog all loaded more highly on the Fatigue/Mixed factor than on the Neurological/Psychiatric factor (which was more characterized by disorientation, visual disturbances, delirium, and altered consciousness). This suggests different types of neurological involvement, potentially reflecting neuroinflammation (the Fatigue/Mixed factor) and encephalitis (the Neurological/Psychiatric factor), respectively. It is of note then that both these factors independently predicted subjective cognitive problems. Both inflammation and encephalitis have been proposed as mechanisms through which COVID-19 may impact the brain (Bougakov et al., 2021) and the presence of indications of neuro-inflammation have been found in post-mortem studies (Matschke et al., 2020). It will be an important next step in the investigation to explore whether the neurological and (possible) inflammatory symptom factors explain variance in performance in cognitive tests.

Participants’ experience of ongoing Neurological, Cardiopulmonary/Fatigue, Gastrointestinal/Autoimmune, Mood and Appetite Loss symptom factors all predicted current cognitive symptoms, together explaining around over 55% of variance. Unlike the initial symptom factors, the vast majority of neurological symptoms were contained within the Neurological factor for ongoing symptoms, with only headache and dizziness loading more strongly into the Gastrointestinal/Autoimmune factor. This latter factor was instead more characterized by symptoms associated with systemic illness—potentially endocrine, or reflecting thyroid disruption—including diarrhea, hot flushes and body pains. An additional predictor here was Cardiopulmonary/Fatigue symptoms, a factor which was quite narrowly characterized by symptoms associated with breathing difficulties. Alone, the Gastrointestinal/Autoimmune and Cardiopulmonary/Fatigue factors explained a large proportion of the variance (36%), suggesting these were the biggest contributor to individual differences in cognitive symptoms. These findings suggest that the symptoms linked with cognitive issues are not so specifically neurological as during the initial illness, but may also incorporate problems with heart and lung function (potentially implying hypoxia, which can induce hypoxic/anoxic-related encephalopathy; Guo et al., 2020) and with other ongoing ill health that is harder to label (resembling symptoms of the menopause, Crohn’s disease, hypothyroidism, and a number of other conditions), but may imply systemic inflammation. Again, these associations align with previous findings, in which cardiopulmonary and cognitive systems clustered in the same factor (Ziauddeen et al., 2021).

In terms of current symptoms, the Cardiopulmonary/Fatigue factor again emerged as a significant predictor, this time paired with Neurological and Appetite Loss symptom factors and explaining nearly 50% of variance. It is potentially notable that both the cognitive and Cardiopulmonary/Fatigue factors showed positive correlation with length of illness, suggesting either that the same disease process underpinning both increases in severity over time, or that the relationship between the two may be the result of both being symptoms more commonly still experienced in those with longer-lasting illness. Longitudinal investigation within individuals would be necessary to disambiguate this.

### Impact of Long COVID

Of those experiencing Long COVID, more than half (and 75% of those with severe symptoms) reported long periods unable to work due to illness. These findings chime with evidence from other studies on Long COVID (e.g., Davis et al., 2021; Ziauddeen et al., 2021). Notably, Davis et al. (2021) found that in their sample 86% of participants reported that it was the cognitive dysfunction *in particular* that was impacting their work (30% severely so). The reported experiences of those with Long COVID—many of whom were at least 6 months into their illness at the time of completing the study—suggest that in addition to broader economic challenges associated with the pandemic, society will face a long “tail” of workforce morbidity. It is thus of great importance—not just for individuals but for society—to be able to prevent, predict, identify and treat issues associated with Long COVID, and including treatment for cognitive symptoms as part of this policy.

A major roadblock to progress in management and treatment of Long COVID is that clinicians do not have the appropriate information or experience. A significant number (over 50% of those with severe symptoms) of our sample reported struggling to get medical professionals to take their symptoms seriously. Part of this issue will be the nature of the symptoms experienced. Patients whose symptoms cannot be, or are not routinely, clinically measured (such as cognitive symptoms; Kaduszkiewicz et al., 2010) are at greater risk of “testimonial injustice”—that is, having their illness dismissed by medical professionals (De Jesus et al., 2021). The novel and heterogenous nature of Long COVID also provides a particular challenge for clinicians dealing with complex and undifferentiated presentations and “medically unexplained symptoms” (Davidson and Menkes, 2021). The data presented here demonstrate that cognitive difficulties reported by patients can be predicted by severity and pattern of symptoms during the initial stages of infection, and during the ongoing illness. These findings should provide the foundation for clinicians to assess the risk of long-term (6 months +) cognitive difficulties, as well as for researchers to investigate the underlying mechanism driving these deficits. In our next paper, we will explore the association between general and cognitive symptoms and performance on cognitive tasks, with the aim of establishing whether self-reported cognitive issues translate into “objective” deficits on cognitive evaluations.

Some have argued that cognitive changes following COVID-19 infection may reflect changes related to experience of lockdown or social isolation (perhaps via development of depression or anxiety). There is indeed some evidence that pandemic-related changes in lifestyle impact cognition (e.g., Fiorenzato et al., 2021; Okely et al., 2021). However, many of these studies did not record COVID-19 infection history (Okely et al., 2021; Smirni et al., 2021) so it is difficult to ascertain to what degree these findings may have been related to COVID-19 infection. One study that did control for this (Fiorenzato et al., 2021) identified significant declines in self-reported attention and executive function, however, showed reduced reports of forgetfulness compared with pre-lockdown. Our results show that, compared to individuals who experienced a (probable) non-COVID-19 illness during the pandemic, those with suspected or confirmed COVID-19 infection experienced greater levels of fatigue, difficulty concentrating, brain fog, tip-of-the-tongue (ToT) word finding problems and semantic disfluency, but did not differ in levels of anxiety and depression. Meanwhile there was little difference between those that did and did not have biological confirmation of their COVID-19 infection. This strongly suggests that self-reported cognitive deficits reported in our sample are associated with COVID-19 infection, rather than the experience of illness, or pandemic more generally.

### Limitations and Future Research

While the findings of this study are notable, there are a number of limitations in design and execution which warrant caution in interpreting the results.

Being unable to bring participants into the lab for clinical assessment, this study relied on online retrospective self-report of symptoms sometimes experienced some months previously. We thus must be cognizant of potential issues of misremembering and that questionnaires may not have been completed in an environment conducive to concentration and reflection. The manner of reporting symptoms differed between different reporting times, with a longer list and more reporting options (reflecting both severity and regularity) for the “ongoing” period. In particular, our binary present/absent reporting approach for currently experienced symptoms was not able to reflect current severity and did not lend itself to factor analysis. Using the *sum scores by factor* method (Tabachnick et al., 2007; Hair, 2009) to calculate alignment of currently experienced symptoms with the symptom factors got around some of these issues, future studies should keep lists consistent to allow for direct comparison of symptom profiles at the different time points. A similar issue is that symptoms information was not collected for the “No COVID” group, or (in terms of current symptoms) for those that reported having recovered. This would have been highly useful in order to establish the degree to which symptoms (particularly those which might be expected to be exacerbated by lockdowns, such as depression, anxiety, fatigue) were more common in those that had previously experienced COVID-19 than those that had not. It would also be useful to ask both the COVID and No COVID groups about their living situation at the time of completing the study, such as whether lockdown or any social restrictions were taking place and how much these measures were affecting their physical and psychological health. It would also have been useful to assess whether people who reported having “recovered” showed symptomatology similar to the “No COVID” group, or remained distinct.

Due to the intensive performance focus of the current investigation, our study had a relatively smaller sample size than is feasible in an epidemiological cohort. Characterizing the sample, we found that those who had experienced COVID-19 infection—and within these, those with more severe ongoing symptoms—tended to be older and more educated. We do not believe that these features reflect vulnerabilities toward COVID-19 or Long COVID, but rather the biases in our recruitment and target populations. Our sample was recruited from English speaking countries (the United Kingdom, Ireland, United States, Canada, Australia, New Zealand, or South Africa) and the majority were from the United Kingdom, which may not be representative of people from other parts of the world. Where possible, we controlled for age, sex, education, and country of residence, which should mitigate some of these biases, however, these sampling discrepancies should be kept in mind. We furthermore specifically targeted our recruitment to those self-identifying as experiencing Long COVID, and we advertised the study as investigating memory and cognition in this group. Our sample may thus have been biased toward those individuals with more severe symptoms and cognitive symptoms in particular (as these individuals may be more motivated to take part). Overrepresentation of Long COVID sufferers is not a serious issue outside of prevalence studies, however, our reported rates of cognitive symptoms within the Long COVID cohort should be treated with caution. It is reassuring, however, that the figures for these symptoms within our cohort are comparable to those seen in much larger studies not explicitly investigating cognition (e.g., Davis et al., 2021; Ziauddeen et al., 2021).

Finally, much of the analysis in this study was necessarily exploratory, as too little was known at the time of study design to form many clear hypotheses. To handle this, multiple comparisons were conducted, for which the alpha adjustments entailed that only the very strongest effects survived at conventional statistical thresholds. This high type 2 error rate means that it is likely that more than just these findings would be confirmed on replication, and because a stated aim of this study was to generate hypotheses that could be tested in later, more targeted research, we have additionally reported the uncorrected results. Similarly, in terms of investigating symptom profiles, we did not aim to present a “definitive” set of factors, but to provide stratifiers and covariates for future analysis, particularly of cognitive test performance, and changes over time. While this study is not able to identify a specific mechanism, it may be able to lay the groundwork with sufficient breadth and detail to inform future mechanistic investigation.

## Conclusion

The COVID and Cognition study is a cross-sectional/longitudinal study assessing symptoms, experiences and cognition in those that have experience COVID-19 infection. Here we present the first analysis in this cohort, characterizing the sample and investigating symptom profiles and cognitive symptoms in particular. We find that particular symptom-profiles—particularly neurological symptoms—during both the initial infection and ongoing illness were predictive of experience of cognitive dysfunction. The symptoms and experiences reported by our sample appear to closely resemble those reported in previous work on Long COVID (e.g., Davis et al., 2021; Ziauddeen et al., 2021) which suggests that our, smaller, sample might be generally representative of the larger Long COVID patient community. The participants in this study are being followed up over the course of the next 1–2 years, and it is hoped that future publications with this sample will provide valuable information as to the time-course of this illness.

The severity of the impact of “Long COVID” on everyday function and employment reported in our sample appear to reflect previous studies (e.g., Davis et al., 2021) and is notable, particularly given the large proportion of healthcare and education staff in our sample. All of these issues should be of interest to policy makers, particularly when considering the extent to which large case numbers should be a concern in the context of reduced hospitalizations and deaths due to vaccination. While we do not yet know the impact of vaccination on Long COVID numbers, there are reasons to believe that high levels of infection among relatively young, otherwise healthy individuals may translate into considerable long-term workforce morbidity.

## Data Availability Statement

The raw data supporting the conclusions of this article will be made available by the authors, without undue reservation.

## Ethics Statement

The studies involving human participants were reviewed and approved by the Psychology Research Ethics Committee, University of Cambridge. The patients/participants provided their written informed consent to participate in this study.

## Author Contributions

LCh and PG designed the study. PG, SY, RL, AS, AB, LCu, and LCh recruited and collected data. PG, AB, SY, MH, and LCh analyzed the data. LCh, PG, SY, and AB wrote the manuscript. All authors contributed to the article and approved the submitted version.

## Conflict of Interest

The authors declare that the research was conducted in the absence of any commercial or financial relationships that could be construed as a potential conflict of interest.

## Publisher’s Note

All claims expressed in this article are solely those of the authors and do not necessarily represent those of their affiliated organizations, or those of the publisher, the editors and the reviewers. Any product that may be evaluated in this article, or claim that may be made by its manufacturer, is not guaranteed or endorsed by the publisher.

## Footnotes

1
www.gorilla.sc
